# INVASIVE SPECIES: Palletable Change in Canada

**DOI:** 10.1289/ehp.117-a489

**Published:** 2009-11

**Authors:** Tim Lougheed

**Affiliations:** **Tim Lougheed** has worked as a freelance writer in Ottawa, Canada, since 1991. A past president of the Canadian Science Writers’ Association, he covers a broad range of topics in science, technology, medicine, and education

Canadian border authorities are no longer accepting crates, pallets, and other solid wood packaging materials from China unless they are stamped with an internationally accepted mark confirming the material has been treated to remove invasive species that might be present in the wood. Cyndee Todgham Cherniak, a counsel in international trade law with Canadian legal firm Lang Michener, says, “[Invasive species in packaging materials] is not a new issue, but I think we’re taking the issue more seriously now than we have before, because pests are being met at the border, and border officers are seeing bugs they’ve never seen before.”

One such arrival is the emerald ash borer (*Agrilus planipennis*), an Asian native suspected to have originally hitched a ride into North America in wood packaging exported from that region. The Canadian Food Inspection Agency (CFIA)—which sets policy regarding the importation of food, plants, animals, and related products into Canada—says the emerald ash borer does not itself pose a direct threat to human health. However, the loss of trees led by the invasive insect represents a lost resource that naturally moderates urban temperatures and improves air quality by absorbing pollutants such as particulate matter, ozone, sulfur dioxide, and nitrogen dioxide. Since it was first detected in 2002 along the U.S.–Canada border flanked by the cities of Detroit and Windsor, the emerald ash borer has been officially identified in 13 U.S. Midwest states, in addition to Ontario and Quebec, wiping out tens of millions of trees along the way, according to the U.S. Department of Agriculture (USDA).

Prior to implementation of the new requirement in September 2009, solid wood packaging from China was allowed into Canada without standardized markings as long as it was accompanied by a “phytosanitary certificate” indicating the wood had been subjected to specific amounts of heating or fumigation with methyl bromide. These techniques became widely standardized in 2002 under the International Plant Protection Convention (IPPC), a treaty that includes nearly 200 member governments under the United Nations Food and Agriculture Organization.

A notice from the Canada Border Services Agency, which enforces CFIA policies at the border, states the CFIA changed the policy “due to high rates of non-compliance from China.” Import agencies commenting on the issue state the difficulty more bluntly as “counterfeit certificates.”

But Julia Dunlop, a forestry specialist with the CFIA, points out that although the Chinese have the option of offering both types of certification, “they are officially suggesting that we recognize only the mark.” Dunlop adds that the move reveals how seriously China wants to be taken in terms of adhering to international standards for packaging among nations belonging to the IPPC.

Those requirements set strict parameters for the mark so it can be readily assessed by border officials: it must at minimum include the IPPC symbol for treated wood packaging materials along with predetermined identification codes for the country of origin, the manufacturing facility, and the treatment applied. Marks must be legible, permanent, non-transferable, and visible on at least two opposite sides of the article being certified. Moreover, the colors red or orange are prohibited to avoid confusion with shipments of dangerous goods. The USDA Animal and Plant Health Inspection Service made the mark mandatory on U.S.-bound wood packaging in 2005.

While it is only logical for U.S. and Canadian authorities to begin demanding more stringent proof that such packaging has been purged of invasive insects, Todgham Cherniak insists it makes sense for the Chinese to appeal to a third-party organization like the IPPC for that proof. “We have to trust each other’s inspection agencies,” she says, arguing that participation in the IPPC opens up the operation of these national organizations to their counterparts in other member states. “You have to get to know the inspection agency, become comfortable, and do a thorough investigation to see whether it has up-to-date equipment and does a good job.”

In this way, she concludes, those same countries can be seen to offer environmental protection at home and abroad without incurring an economic penalty. “We’re protecting our people,” she says. “We are protecting our domestic market. But we’re going to facilitate trade. This is the best way possible: having mutual recognition.”

